# Frontline nilotinib in patients with chronic myeloid leukemia in chronic phase: results from the European ENEST1st study

**DOI:** 10.1038/leu.2015.270

**Published:** 2015-11-03

**Authors:** A Hochhaus, G Rosti, N C P Cross, J L Steegmann, P le Coutre, G Ossenkoppele, L Petrov, T Masszi, A Hellmann, L Griskevicius, W Wiktor-Jedrzejczak, D Rea, D Coriu, T H Brümmendorf, K Porkka, G Saglio, G Gastl, M C Müller, P Schuld, P Di Matteo, A Pellegrino, L Dezzani, F-X Mahon, M Baccarani, F J Giles

**Affiliations:** 1 Abteilung Hämatologie/Onkologie, Klinik für Innere Medizin II, Universitätsklinikum Jena, Jena, Germany; 2 Department of Hematology and Oncology, University of Bologna, Bologna, Italy; 3 Faculty of Medicine, University of Southampton, Southampton, UK; 4 Hospital Universitario de la Princesa, IIS-IP, Madrid, Spain; 5 Charité—Universitätsmedizin Berlin, Berlin, Germany; 6 VU University Medical Center, Amsterdam, The Netherlands; 7 Ion Chiricuta Institute of Oncology, Cluj, Romania; 8 Department of Haematology and Stem Cell Transplantation, St István and St László Hospital, Budapest, Hungary; 9 Medical University of Gdańsk, Gdańsk, Poland; 10 Vilnius University Hospital Santariskiu Klinikos, Vilnius University, Vilnius, Lithuania; 11 Department of Hematology, Medical University of Warsaw, Warsaw, Poland; 12 Service d'Hématologie Adulte et INSERM UMR1160, Hôpital Saint-Louis, Paris, France; 13 Fundeni Clinical Institute, Bucharest, Romania; 14 Universitätsklinikum RWTH Aachen, Aachen, Germany; 15 Department of Hematology, Helsinki University Hospital Comprehensive Cancer Center, Helsinki, Finland; 16 Division of Internal Medicine & Hematology, University of Turin, Orbassano, Italy; 17 Innere Medizin, Medizinische Universität Innsbruck, Innsbruck, Austria; 18 Universitätsmedizin Mannheim, University of Heidelberg, Mannheim, Germany; 19 Novartis Pharma AG, Basel, Switzerland; 20 Novartis Oncology Region Europe, Origgio, Italy; 21 Laboratoire Hématopoïèse Leucémique et Cible Thérapeutique, Université Victor Ségalen, Bordeaux, France; 22 Northwestern Medicine Developmental Therapeutics Institute, Robert H. Lurie Comprehensive Cancer Center of Northwestern University, Chicago, IL, USA

## Abstract

The Evaluating Nilotinib Efficacy and Safety in Clinical Trials as First-Line Treatment (ENEST1st) study included 1089 patients with newly diagnosed chronic myeloid leukemia in chronic phase. The rate of deep molecular response (MR^4^ (*BCR-ABL1*⩽0.01% on the International Scale or undetectable *BCR-ABL1* with ⩾10 000 *ABL1* transcripts)) at 18 months was evaluated as the primary end point, with molecular responses monitored by the European Treatment and Outcome Study network of standardized laboratories. This analysis was conducted after all patients had completed 24 months of study treatment (80.9% of patients) or discontinued early. In patients with typical *BCR-ABL1* transcripts and ⩽3 months of prior imatinib therapy, 38.4% (404/1052) achieved MR^4^ at 18 months. Six patients (0.6%) developed accelerated or blastic phase, and 13 (1.2%) died. The safety profile of nilotinib was consistent with that of previous studies, although the frequencies of some nilotinib-associated adverse events were lower (for example, rash, 21.4%). Ischemic cardiovascular events occurred in 6.0% of patients. Routine monitoring of lipid and glucose levels was not mandated in the protocol. These results support the use of frontline nilotinib, particularly when achievement of a deep molecular response (a prerequisite for attempting treatment-free remission in clinical trials) is a treatment goal.

## Introduction

Nilotinib is a BCR-ABL1 tyrosine kinase inhibitor approved for the treatment of patients with newly diagnosed Philadelphia chromosome-positive (Ph+) chronic myeloid leukemia in chronic phase (CML-CP) or Ph+ CML in accelerated phase (AP) or CP that is resistant to or intolerant of prior therapy, including imatinib.^1^

Throughout 6 years of follow-up in the pivotal trial of frontline nilotinib vs imatinib for patients with CML-CP (Evaluating Nilotinib Efficacy and Safety in Clinical Trials–Newly Diagnosed Patients (ENESTnd)), nilotinib showed improved efficacy over imatinib, including earlier and deeper molecular responses.^2, 3, 4, 5, 6, 7^ ENESTnd met its primary end point, with statistically significantly higher rates of major molecular response (MMR; *BCR-ABL1*⩽0.1% on the International Scale (IS)) at 12 months with nilotinib 300 mg twice daily (44%) and nilotinib 400 mg twice daily (43%) than with imatinib (22% *P* <0.001 vs either nilotinib arm).^5^ Moreover, progression to AP or blastic phase (BP) tended to be less common with nilotinib; by the 6-year data cutoff, 11 (nominal *P*=0.0661 vs imatinib), 6 (nominal *P*=0.0030 vs imatinib) and 21 patients in the nilotinib 300-mg twice-daily, nilotinib 400-mg twice-daily and imatinib arms, respectively, progressed to AP/BP on study.^6^ Although the total number of deaths on study was similar in the nilotinib 300-mg twice-daily and imatinib arms (by the 6-year data cutoff: nilotinib 300-mg twice-daily, 21; nilotinib 400-mg twice-daily, 11; imatinib, 23), fewer patients in the nilotinib arms than in the imatinib arm died owing to advanced CML (6, 4 and 16, respectively).^6^

The safety profile of nilotinib is distinct from that of imatinib. In ENESTnd, nausea, diarrhea and muscle spasms were the most common adverse events (AEs) reported with imatinib, whereas rash and headache were most common with nilotinib; although not among the most common AEs, cardiovascular events were also more frequent with nilotinib.^3, 4, 5, 6, 7^ In contrast to the trend for fewer deaths with the higher dose of nilotinib vs the lower dose, cardiovascular events were less common with the lower nilotinib dose.^6^

ENEST1st was a phase 3b, multicenter, single-arm, open-label study investigating the efficacy and safety of nilotinib 300 mg twice daily (the recommended starting dose for patients with newly diagnosed CML-CP^1^) in a large population of patients with newly diagnosed Ph+ or Ph− *BCR-ABL1*+ CML-CP. Because achievement of a deep molecular response is a key eligibility criterion for attempting treatment-free remission (TFR) in clinical trials,^8, 9, 10, 11, 12, 13^ ENEST1st was designed with an emphasis on evaluating rates of deep molecular responses achieved with frontline nilotinib. Although the most appropriate depth of response (for example, molecular response 4 (MR^4^; *BCR-ABL1*^IS^⩽0.01%), molecular response 4.5 (MR^4.5^; *BCR-ABL1*^IS^⩽0.0032%) or deeper) at which to attempt TFR remains unknown, TFR following achievement of sustained MR^4^ is being investigated in ongoing studies^12, 13^; thus the ENEST1st primary end point analysis (rate of MR^4^ at 18 months) provides an indication of the proportion of nilotinib-treated patients who could be expected to meet the eligibility requirements for such studies. In addition to evaluating deep molecular responses, ENEST1st was the first study to evaluate the association between baseline European Treatment and Outcome Study (EUTOS) risk score^14^ and nilotinib efficacy and the first study to conduct molecular monitoring using a network of IS-standardized laboratories, with 14 EUTOS laboratories participating. Here the final results of ENEST1st are reported, based on analyses conducted after all patients completed 24 months of study treatment or discontinued early.

## Methods

### Patients, study design and treatments

Adults (aged ⩾18 years) with newly diagnosed (⩽6 months), cytogenetically confirmed Ph+ CML-CP or Ph− *BCR-ABL1*+ CML-CP were eligible for enrollment. World Health Organization performance status ⩽2 was required. Patients previously treated with hydroxyurea for >6 months and imatinib for >3 months were excluded. Patients were also excluded if they had known impairments in cardiac function (including left ventricular ejection fraction <45%, complete left bundle branch block, right bundle branch block plus left anterior hemiblock/bifascicular block, ventricular-paced pacemaker, congenital long QT syndrome, history or presence of clinically significant ventricular or atrial tachyarrhythmia, clinically significant resting bradycardia, QTcF>450 ms, myocardial infarction within the past 12 months or other clinically significant heart disease), history of acute or chronic pancreatitis, impaired gastrointestinal function, concurrent uncontrolled medical conditions that would present unacceptable safety risks or compromise compliance with the protocol, major surgery within the past 2 weeks or not recovered from side effects of surgery or concomitant treatment with medications with the potential to prolong the QT interval or known to be strong inducers or inhibitors of cytochrome P450 3A4.

All enrolled patients were treated with nilotinib at a starting dose of 300 mg twice daily, for up to 24 months. Dose escalation was not allowed. Dose interruptions were recommended for patients with study drug-related, clinically significant, nonhematological, noncardiac grade 2/3 AEs or study drug-related grade 3/4 events related to white blood cells or platelets. Recommendations for treatment resumption/dose reduction were as follows: following the first and second occurrences, resume treatment with nilotinib 600 mg/day upon improvement to grade <2 (nonhematological) or <3 (hematological); following the third occurrence, resume treatment with nilotinib 450 mg/day upon improvement to grade <2 (nonhematological) or <3 (hematological), escalating to 600 mg/day after 1 week; following the fourth occurrence, resume treatment with nilotinib 300 mg/day upon improvement to grade <2 (nonhematological) or <3 (hematological), escalating to 600 mg/day after 1 month (escalation for hematological events only); following the fifth occurrence (or following any study drug-related, clinically significant, nonhematological grade 4 AE), stop treatment and contact the study management committee. For patients with corrected QT interval (QTc) prolongation from 480 to 499 ms, nilotinib dose interruption followed by resumption at 450 mg/day upon improvement to QTc⩽450 ms was recommended; for patients with QTc prolongation to ⩾500 ms or with significant cardiac conduction or rhythm abnormalities, permanent discontinuation of nilotinib was recommended.

### Assessments

*BCR-ABL1* transcript type was determined by multiplex PCR at baseline.^15^ Molecular responses were assessed every 3 months during study treatment using real-time quantitative PCR (RQ-PCR) at designated EUTOS laboratories standardized to the IS. Deep molecular responses were scored in accordance with the EUTOS recommendations in place at the time.^16^ MR^4^ was defined as detectable *BCR-ABL1*^IS^⩽0.01% or undetectable *BCR-ABL1* in samples with ⩾10 000 *ABL1* transcripts. Samples with a mean of <10 000 *ABL1* transcripts, or with a total of<10 000 *ABL1* transcripts in the case of undetectable *BCR-ABL1*, were considered unevaluable for MR^4^. MR^4.5^ was defined as detectable *BCR-ABL1*^IS^ ⩽0.0032% or undetectable *BCR-ABL1* in samples with ⩾32 000 *ABL1* transcripts. Samples with a mean of <32 000 *ABL1* transcripts, or with a total of<32 000 *ABL1* transcripts in the case of undetectable *BCR-ABL1*, were considered unevaluable for MR^4.5^.^16^

Bone marrow cytogenetic assessments were performed ⩽8 weeks before the first dose of nilotinib and at months 3 and 6 and every 6 months thereafter until MMR was achieved or the patient discontinued nilotinib. Cytogenetic assessments were performed and analyzed locally using standard methods; fluorescence *in situ* hybridization analyses were not allowed.

### End points and definitions

The primary end point was the rate of MR^4^ at 18 months. Secondary end points included the rates of complete cytogenetic response (CCyR; 0% Ph+ metaphases), MMR, MR^4^ and MR^4.5^ at and by 12 and 24 months; progression to AP/BP; progression-free survival; overall survival (OS); and safety. Patients who discontinued study treatment early were followed for survival for up to 24 months; data regarding other outcomes (including progression) were not collected after discontinuation of study treatment. Progression-free survival was defined as the time from the first dose of study treatment until documented disease progression or death owing to any cause. OS was defined as the time from the first dose of study treatment until death owing to any cause at any time (including after discontinuation of study treatment). Patients were monitored for AEs throughout study treatment and for up to 28 days following the last dose of study drug. AEs were assessed according to the Common Terminology Criteria for Adverse Events version 4.0.^17^ AE types included in the definition of ischemic cardiovascular events (subdivided into three groups: peripheral artery disease, ischemic heart disease, and ischemic cerebrovascular events) are detailed in Supplementary Table S1.

### Statistical analyses

All patients who received ⩾1 dose of study treatment were included in the intent-to-treat and safety populations. Patients with typical *BCR-ABL1* transcripts (that is, b2a2 and/or b3a2) and ⩽3 months of prior imatinib treatment were included in the molecular analysis population for evaluating molecular response rates (patients with atypical *BCR-ABL1* transcripts were excluded because standard RQ-PCR methodology was not optimized for detection of atypical *BCR-ABL1* transcripts; patients with >3 months of imatinib therapy (a protocol violation) were excluded to be as conservative as possible in analyzing the efficacy of frontline nilotinib by avoiding potential confounding effects of prior imatinib). Patients with typical *BCR-ABL1* transcripts, no prior imatinib exposure and evaluable RQ-PCR assessments at 3 months were included in the landmark analysis population; patients who had already achieved the target response (MMR, MR^4^ or MR^4.5^, respectively) at 3 months were excluded from the landmark analyses of MMR, MR^4^ and MR^4.5^ rates over time. Patients with Ph+ metaphases detected at screening or without evaluable cytogenetic analyses at screening but with Ph+ metaphases detected at a later time point were included in the cytogenetic analysis population for evaluating rates of CCyR (patients without confirmed Ph+ disease were unevaluable for CyR and were excluded from the analysis of CCyR rates).

For calculation of response rates ‘at' a designated time point, patients were considered responders only if an assessment at that time point showed achievement of the response. Response rates ‘by' a designated time point were calculated as cumulative response rates, counting all patients with a response detected at or before the specified time point as responders. All response rates were calculated as raw proportions. Rates of freedom from progression to AP/BP on treatment and OS were estimated using Kaplan–Meier product limit estimates according to intent-to-treat principles.

### Ethics

ENEST1st was conducted in accordance with the International Conference on Harmonisation Harmonised Tripartite Guidelines for Good Clinical Practice, the Declaration of Helsinki and applicable local regulations. Eligible patients were included only after providing written consent and in accordance with local laws and regulations. The protocol and informed consent forms were reviewed and approved by an institutional review board, independent ethics committee or research ethics board before study start at each participating institution. ENEST1st was registered in the EU Clinical Trials Registry (2009-017775-19) and ClinicalTrials.gov (NCT01061177).

## Results

### Patients and treatment exposure

**Figure 1:**
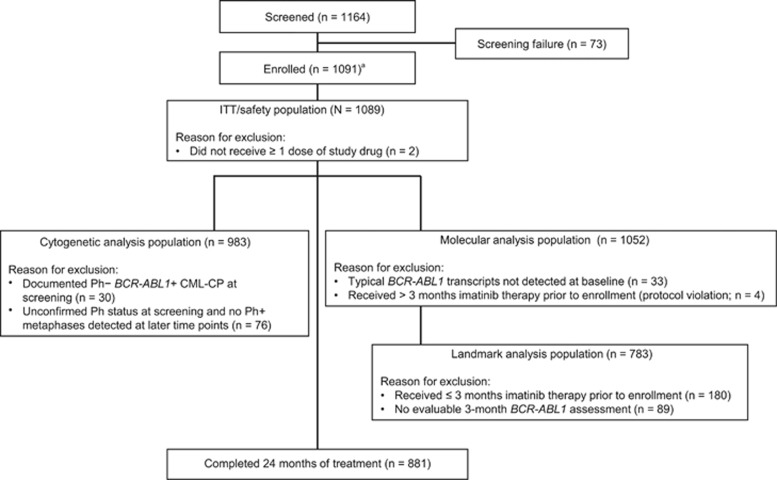
Analysis populations. ^a^The original target enrollment of *N*=806 (determined using an approximation of a normal distribution to achieve a precision of 3.3% for the 95% CI of the primary end point, assuming an MR^4^ rate of 25% at 18 months, a discontinuation rate of 15% and a Philadelphia chromosome-negative and/or atypical *BCR-ABL1* transcript prevalence of 5%) was increased to allow a more robust analysis of the study's exploratory results. ITT, intent to treat.

From 2010 to 2012, 1164 patients were screened, 1091 were enrolled from 307 sites in 26 European countries (Supplementary Figure S1; Supplementary Table S2) and 1089 received ⩾1 dose of nilotinib 300 mg twice daily (Figure 1). The median age of treated patients was 53 years (Table 1). The median time since diagnosis was 0.9 months, and 70.3% of patients (*n*=766) had received prior treatment for CML (hydroxyurea, 52.9% (*n*=576); imatinib, 17.3% (*n*=188); other, 0.2% (*n*=2)). EUTOS risk scores were low and high in 82.6% (*n*=900) and 8.6% (*n*=94) of patients, respectively; Sokal risk scores were low, intermediate and high in 34.6% (*n*=377), 37.5% (*n*=408) and 18.1% (*n*=197), respectively. EUTOS and Sokal risk scores could not be calculated in 8.7% (*n*=95) and 9.8% (*n*=107) of patients, respectively, owing to missing baseline data for ⩾1 parameter required for the calculation.

A total of 80.9% of patients (*n*=881) completed 24 months of study treatment, and 19.1% (*n*=208) discontinued before 24 months (Table 2). Dose changes or interruptions occurred in 45.2% of patients (*n*=492), including 36.7% (*n*=400) with changes or interruptions owing to AEs or laboratory abnormalities.

### Molecular and cytogenetic responses

**Figure 2:**
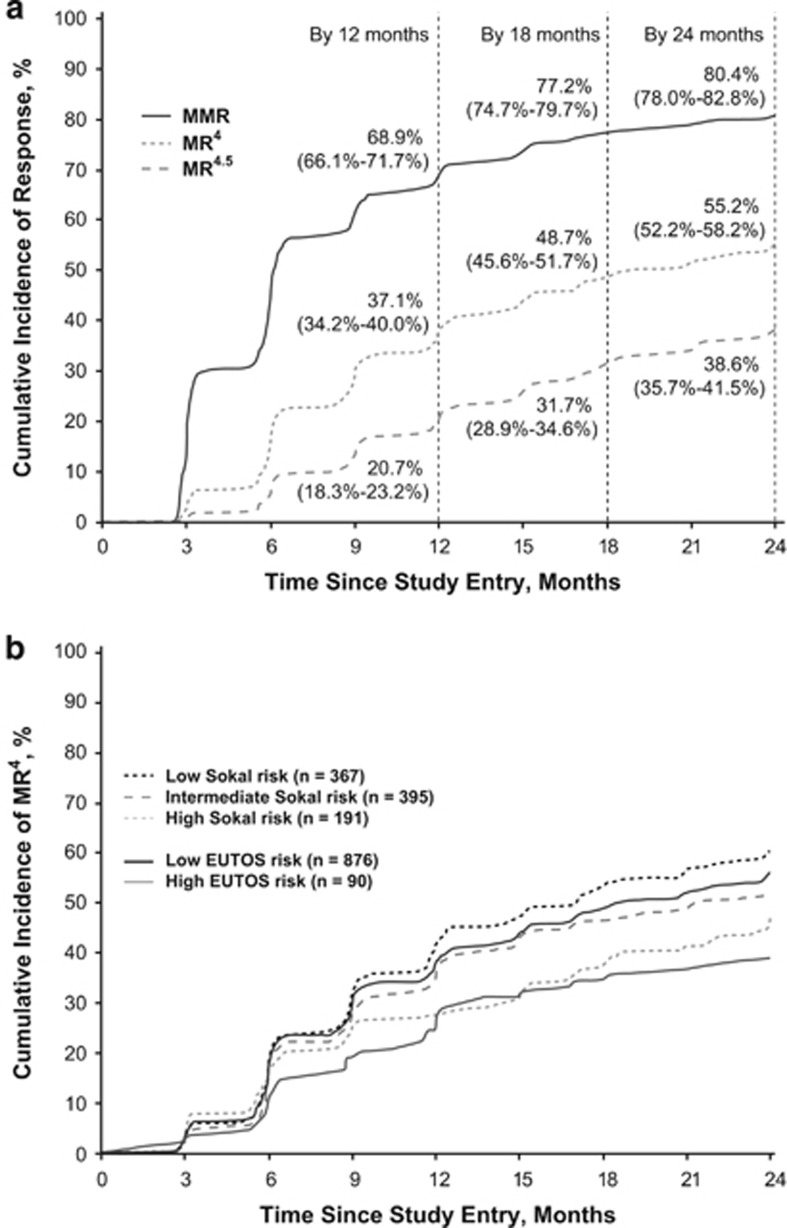
Cumulative molecular response rates. Raw cumulative incidence (95% CI) of (**a**) MMR (*BCR-ABL1*^IS^⩽0.1%), MR^4^ (*BCR-ABL1*^IS^⩽0.01%) and MR^4.5^ (*BCR-ABL1*^IS^⩽0.0032%) in the molecular analysis population (*n*=1052) and (**b**) MR^4^ according to European Treatment and Outcome Study (EUTOS) and Sokal risk scores at diagnosis.

Among patients in the molecular analysis population (*n*=1052), the rate of MR^4^ at 18 months was 38.4% (95% confidence interval (CI), 35.5–41.3% *n*=404). Rates of MMR, MR^4^ and MR^4.5^ at 3, 12, 18 and 24 months are listed in Table 3. Cumulative rates of MMR, MR^4^ and MR^4.5^ by 24 months were 80.4% (*n*=846), 55.2% (*n*=581) and 38.6% (*n*=406), respectively (Figure 2a). Among patients remaining on study at 18 and 24 months, 13.7% (121/886) and 9.2% (79/855) had not achieved MMR by 18 or 24 months, respectively.

Patients with low EUTOS or Sokal risk scores were more likely than those with higher scores to achieve MR^4^ (Figure 2b). At 24 months, MR^4^ rates (95% CI) among patients with low and high EUTOS risk scores were 41.4% (38.2–44.7% 363/876) and 27.8% (18.5–37.0% 25/90), respectively; MR^4^ rates (95% CI) at 24 months were 44.7% (39.6–49.8% 164/367), 39.7% (34.9–44.6% 157/395), and 31.4% (24.8–38.0% 60/191) among patients with low intermediate and high Sokal risk scores, respectively.

**Figure 3:**
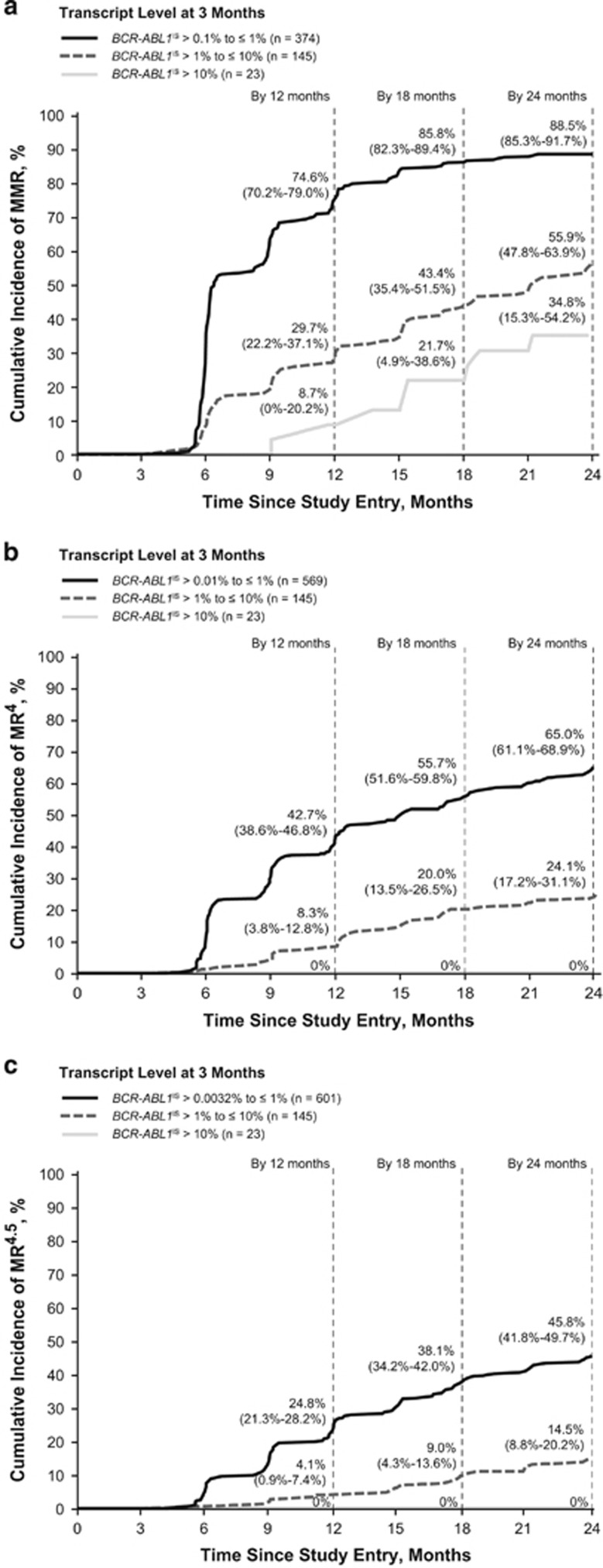
Cumulative molecular response rates according to 3-month *BCR-ABL1*^IS^. (**a**) Raw cumulative rates (95% CI) of MMR (*BCR-ABL1*^IS^⩽0.1%), (**b**) MR^4^ (*BCR-ABL1*^IS^⩽0.01%) and (**c**) MR^4.5^ (*BCR-ABL1*^IS^⩽0.0032%) among patients in the landmark analysis population (*n*=783) with *BCR-ABL1*^IS^⩽1%, >1–⩽10% and >10% at 3 months. Patients who had already achieved MMR, MR^4^ or MR^4.5^, respectively, at 3 months were excluded from the landmark analyses of MMR, MR^4^ and MR^4.5^ rates over time.

Among patients in the molecular analysis population with no prior imatinib exposure (*n*=872), 81.2% (*n*=708), 55.5% (*n*=484) and 37.8% (*n*=330) achieved MMR, MR^4^ and MR^4.5^, respectively, by 24 months. Of patients in this subpopulation with evaluable 3-month RQ-PCR assessments (*n*=783; landmark analysis population), 78.5% (*n*=615), 18.5% (*n*=145) and 2.9% (*n*=23) had *BCR-ABL1*^IS^⩽1%, *BCR-ABL1*^IS^>1–⩽10% and *BCR-ABL1*^IS^>10%, respectively, at 3 months. Rates of MMR, MR^4^ and MR^4.5^ by 24 months were the highest among patients with *BCR-ABL1*^IS^⩽1% at 3 months (excluding those with MMR (*n*=241), MR^4^ (*n*=46) or MR^4.5^ (*n*=14), respectively, at 3 months) and the lowest among patients with *BCR-ABL1*^IS^>10% at 3 months (Figure 3).

Patients in the intent-to-treat population with documented Ph− *BCR-ABL1*+ disease at screening (*n*=30) or with unconfirmed Ph status at screening and no Ph+ metaphases detected at later time points (*n*=76) were excluded from the cytogenetic analysis population. Among the remaining patients (*n*=983), 67.3% (95% CI, 64.4–70.3% *n*=662) achieved CCyR by 6 months and 82.5% (95% CI, 80.1–84.9% *n*=811) achieved CCyR by 12 months.

### Survival and progression

Estimated OS at 24 months was 98.9% (95% CI, 98.0–99.4%), with 13 deaths reported on study (⩽24 months after first dose of study treatment). Four patients died ⩽28 days after the last dose of study treatment/month 24 evaluation (one each due to pulmonary embolism, congestive heart failure, thrombocytopenia and infection), and nine patients died >28 days after the last dose of study treatment/month 24 evaluation (three each due to infections and secondary cancers and one each due to cerebral infarction, heart failure and CML progression). Six patients (0.6%) progressed to AP (*n*=3) or BP (*n*=3) on treatment, none of whom died on study. At 24 months, the estimated rate of freedom from progression to AP/BP on treatment was 99.4% (95% CI, 98.7–99.7%).

### Safety

Rash, pruritus and headache were the most common nonhematological AEs, reported in 21.4% (*n*=233), 16.5% (*n*=180) and 15.2% (*n*=166) of patients, respectively (Table 4). Pancreatitis, hepatotoxicity and fluid retention occurred in 1.0% (*n*=11; grade 3/4, 0.6%), 1.4% (*n*=15; grade 3/4, 0.4%) and 11.8% (*n*=129; grade 3/4, 0.8%) of patients, respectively. Pleural effusion occurred in 0.6% (*n*=7; grade 3/4, 0.2%) of patients; 1 patient with grade 3 pleural effusion was treated with imatinib before enrollment (imatinib duration, 53 days). No patient was diagnosed with pulmonary hypertension. Arrhythmia and supraventricular arrhythmia were reported in 0.6% (*n*=6) and 0.1% (*n*=1) of patients, respectively (including grade 3/4 arrhythmia in 1 patient). Congestive heart failure was reported in 0.3% of patients (*n*=3; all grade 3). Ischemic cardiovascular events occurred in 6.0% of patients (*n*=65; grade 3/4, 3.5%), including peripheral artery disease in 1.9% (*n*=21; grade 3/4, 0.7%), ischemic heart disease in 3.4% (*n*=37; grade 3/4, 2.2%) and ischemic cerebrovascular events in 0.8% (*n*=9; grade 3/4, 0.6%). Four patients died due to ischemic cardiovascular events, one each due to congestive heart failure, cerebral infarction, heart failure and ischemic stroke (the death due to ischemic stroke occurred >24 months after first dose of study drug; therefore, it was not considered in the OS analysis).

Grade 3/4 thrombocytopenia and neutropenia were reported in 6.0% (*n*=65) and 4.8% (*n*=52) of patients, respectively (Table 5). Grade 3/4 biochemical abnormalities of decreased phosphate level and increased lipase activity occurred in 14.3% (*n*=156) and 7.2% (*n*=78) of patients, respectively.

Because routine monitoring of lipid and glucose levels was not mandated in the study protocol, the frequencies of hypercholesterolemia, hyperglycemia and diabetes mellitus on study could not be evaluated; however, AEs of hypercholesterolemia, hyperglycemia and diabetes mellitus were spontaneously reported in 3.0% (*n*=33), 3.3% (*n*=36) and 1.2% (*n*=13) of patients, respectively.

## Discussion

ENEST1st was the first study to investigate deep molecular response as the primary end point. Results from this study confirm the high rates of deep responses achieved with frontline nilotinib; 38.4% of patients in the molecular analysis population achieved the primary end point of MR^4^ at 18 months, and by 24 months, 55.2% achieved MR^4^ and 38.6% achieved MR^4.5^. Although the most appropriate molecular response threshold for attempting TFR remains under investigation, the feasibility of TFR following achievement of a sustained, deep molecular response on nilotinib has been demonstrated,^11^ and ongoing studies are evaluating TFR in patients with sustained MR^4^ or MR^4.5^ on nilotinib.^12, 13^ The high rates of MR^4^ and MR^4.5^ in ENEST1st suggest that many patients treated with frontline nilotinib may be able to achieve the level of response necessary to qualify for such studies.

In addition to enabling the possibility of TFR, deep molecular responses have been linked to improved patient outcomes, including prolonged survival and decreased risk of disease progression.^18, 19, 20^ In a recent study of patients treated with frontline imatinib, those with MR^4^ at 24 months had a 0% estimated incidence of leukemia-related death by 6 years vs 7% for patients without MR^4^ at 24 months (*P*=0.004).^21^ Although many imatinib-treated patients eventually attain deep molecular responses with long-term therapy,^21, 22^ results from ENESTnd demonstrated that patients achieved faster and higher rates of such responses with frontline nilotinib vs imatinib.^3, 4, 5, 6, 7^

The use of the EUTOS laboratory network to assess molecular responses in ENEST1st demonstrated the feasibility of noncentralized, regional molecular monitoring, provided that standardized and sensitive assays are used and definitions are harmonized.^16^ Through the work of the EUTOS Study Group, recommendations for standardized scoring of deep molecular responses have been developed, which will allow for more consistent patient management across local and regional treatment centers.^23^ As ENEST1st was the first study to assess nilotinib efficacy according to EUTOS risk scores, these results validate the use of this newer risk score for assessing patient prognosis at baseline.^14^ Importantly, data from a EUTOS population-based registry of 2904 patients with CML (94.3% with CML-CP) suggest that the age, sex and EUTOS risk score distribution of patients in ENEST1st were generally similar to what is observed in routine clinical practice.^24^

Patients in ENEST1st were slightly older than those in the nilotinib 300-mg twice-daily arm of ENESTnd (median age of 53 years in ENEST1st vs 47 years in ENESTnd); however, the distribution of Sokal risk scores was similar (72% of patients in each study had low or intermediate Sokal risk scores).^5^ Nonetheless, molecular response rates were higher in ENEST1st than in ENESTnd; in ENEST1st, 81, 56 and 38% of patients without prior imatinib exposure (the subpopulation most comparable to patients in ENESTnd) achieved MMR, MR^4^ and MR^4.5^, respectively, by 2 years, compared with 71, 39 and 25% of patients, respectively, in the nilotinib 300-mg twice-daily arm of ENESTnd.^4^ This difference may be due in part to improvements in the management of nilotinib-treated patients, that is, a learning effect in the period since ENESTnd was initiated (in 2007).^5^ For example, whereas the ENESTnd protocol called for dose reduction to nilotinib 400 mg once daily after the first or second occurrence of study drug-related AEs, and permanent discontinuation after the next occurrence,^5^ new dose reduction guidelines in ENEST1st allowed for stepwise or temporary dose reductions to nilotinib 450 or 300 mg daily in patients with recurrent AEs, which may have enabled patients to receive a more optimal nilotinib dose. In the nilotinib 300-mg twice-daily arm of ENESTnd, 55% of patients had dose interruptions/reductions owing to AEs by the 2-year data cutoff, and the median dose intensity was 594 mg/day (25th–75th percentile, 553–600 mg/day).^4^ By comparison, 36.7% of patients in ENEST1st had dose changes or interruptions owing to AEs/laboratory abnormalities, and the 25th percentile for dose intensity was 35 mg/day higher than that in ENESTnd.

Consistent with prior studies,^2, 25, 26, 27^ ENEST1st demonstrated the importance of early molecular response to frontline treatment. Patients with *BCR-ABL1*^IS^⩽1% at 3 months achieved the highest rates of response at later time points, whereas no patient with *BCR-ABL1*^IS^>10% at 3 months achieved MR^4^ by 24 months. Because of the known association between early molecular response and long-term outcomes, both the European LeukemiaNet and the National Comprehensive Cancer Network recommend *BCR-ABL1*^IS^⩽10% as a target response at 3 months.^28, 29^ Nearly all patients (97%) in the ENEST1st landmark analysis population achieved this target, consistent with results from ENESTnd.^2^ Although achievement of *BCR-ABL1*^IS^⩽1% at 3 months is not a designated treatment goal in current CML guidelines,^28, 29^ the results reported here are consistent with those reported elsewhere in demonstrating the value of this landmark for predicting future achievement of deep molecular response.^2, 18^ In addition to absolute *BCR-ABL1*^IS^ levels at 3 months, some studies have shown that the rate of decline in *BCR-ABL1* levels early during treatment may provide a further indication of expected long-term outcomes.^30, 31, 32^ Branford *et al.*^31^ demonstrated that, among patients with *BCR-ABL1*^IS^>10% at 3 months, those with a *BCR-ABL1* halving time of >76 days had poorer long-term outcomes than those with a halving time of ⩽76 days, while Hanfstein *et al.*^30^ showed that the reduction in *BCR-ABL1* transcript levels at 3 months relative to each patient's individual baseline level was a significant predictor of survival.

Safety results from ENEST1st were similar to those of ENESTnd,^3, 4, 5, 6, 7^ with no new safety signals identified. However, although patients in ENEST1st were older than patients in ENESTnd,^5^ the frequencies of some AEs, including rash and grade 3/4 thrombocytopenia and neutropenia, were lower in ENEST1st (reported frequencies in the nilotinib 300-mg twice-daily arm of ENESTnd by the 2-year data cutoff: rash, 41% grade 3/4 thrombocytopenia, 10% grade 3/4 neutropenia, 12%).^4^ The relatively low frequencies of these common AE types further supports the notion that patients in ENEST1st were more optimally managed than patients in earlier studies—a frequent occurrence as experience is gained with cancer therapies.^33, 34, 35^ Although baseline cardiovascular risk factors were not collected in ENEST1st, the observed frequency of ischemic cardiovascular events (6%) was comparable to what has been previously reported for nilotinib-treated patients in ENESTnd.^6^ However, as some patients in ENESTnd experienced cardiovascular events at later time points (beyond the first 2 years of treatment),^6^ it is possible that the total frequency of such events in ENEST1st would increase with longer follow-up.

Nilotinib treatment is known to be associated with certain biochemical abnormalities, including hyperglycemia and hypercholesterolemia.^3, 4, 5, 6, 7^ Because glucose and lipid monitoring was not mandated in the ENEST1st protocol, the frequencies of glucose and lipid abnormalities are unknown; the rates of spontaneously reported hyperglycemia, hypercholesterolemia and diabetes mellitus likely underestimate the true frequency of these events. For patients treated with nilotinib, it is recommended that lipid and glucose levels be monitored before initiating treatment and during treatment, and all cardiovascular risk factors should be monitored and actively managed according to standard guidelines.^1^

The high rates of response and very low rate of progression to AP/BP (0.6%) in ENEST1st demonstrate the efficacy of frontline nilotinib for the majority of patients; indeed, among patients remaining on treatment at 24 months, only 9.2% had not achieved MMR. However, 19.1% of patients discontinued treatment before the 24-month assessment, clearly indicating that further improvements in the frontline management of patients with CML-CP are needed. Overall, results from ENEST1st, including those reported here and data from 11 substudies directed by national and international study groups, provide further support for the use of nilotinib 300 mg twice daily as a frontline treatment option for patients with newly diagnosed CML-CP.

## Figures and Tables

**Table 1:** Demographic and baseline characteristics (ITT population; *N*=1089)

	N=*1089*
Median age (range), years	53 (18–91)

*Sex, n (%)*	
Male	642 (59.0)
Female	447 (41.0)
	
White, *n* (%)	1045 (96.0)
Median time since diagnosis (range), months	0.9 (<0.1–6.6)^a^
	
*Prior treatment for CML, n (%)*	766 (70.3)
Imatinib	188 (17.3)
Hydroxyurea	576 (52.9)
Other	2 (0.2)
	
Median prior treatment duration (range), months^b^	0.9 (0.1–7.6)^a^

*Cytogenetic classification, n (%)*	
Ph+	983 (90.3)
Ph−/BCR-ABL1+	30 (2.8)
Unknown	76 (7.0)

*Transcript type, n (%)*	
b2a2 and/or b3a2	1056 (97.0)
Other^c^	16 (1.5)
Inadequate sample, not evaluated or not reported	17 (1.6)

*EUTOS risk, n (%)*	
Low	900 (82.6)
High	94 (8.6)
Missing^d^	95 (8.7)

*Sokal risk, n (%)*	
Low	377 (34.6)
Intermediate	408 (37.5)
High	197 (18.1)
Missing^d^	107 (9.8)

Abbreviations: CML, chronic myeloid leukemia; EUTOS, European Treatment and Outcome Study; ITT, intent to treat; Ph, Philadelphia chromosome.

a One patient was pretreated with hydroxyurea while awaiting confirmation of CML diagnosis and, therefore, had a longer prior treatment duration than time since diagnosis. Four patients with >3 months of prior exposure to imatinib were excluded from the analysis of median and range; for these 4 patients, prior treatment durations were 93, 114, 117 and ⩾1089 days, respectively.

b Among patients with prior CML treatments.

c Including e1a2, e19a2, e14a3, e18a2, e8a2 and e13a3.

d EUTOS and/or Sokal risk scores could not be calculated for patients with missing data for baseline parameters required for the calculation of these scores.

**Table 2:** Patient disposition and treatment exposure (safety population; *N*=1089)

*Patients, n (%)*	N=*1089*
Completed ⩾24 months of treatment	881 (80.9)
	
*Discontinued treatment*^a^	208 (19.1)
Adverse event	117 (10.7)
Withdrew consent^b^	27 (2.5)
Disease progression/treatment failure	17 (1.6)
Abnormal laboratory value	6 (0.6)
Abnormal test procedure result	4 (0.4)
Other^c^	37 (3.4)
	
Median duration of exposure (25th–75th percentile), days^d^	722 (691–734)
Median nilotinib dose intensity (25th–75th percentile), mg/day^e^	600 (588–600)
	
*Patients with dose interruption, n (%)*	387 (35.5)
Median number of interruptions per patient (25th–75th percentile)	1 (1–2)
Median duration of interruption (25th–75th percentile), days	14 (7–30)
	
Patients with dose reduction, *n* (%)	321 (29.5)

*Maximal dose reduction, n (%)*	
To 450 mg/day	22 (2.0)
To 400 mg/day	2 (0.2)
To 300 mg/day	263 (24.2)
To 150 mg/day	34 (3.1)
	
Total patients with dose change/interruption, *n* (%)	492 (45.2)

*Reason for dose change/interruption*	
Adverse event/laboratory abnormality	400 (36.7)
Dosing error^f^	123 (11.3)
Scheduling conflict^f^	72 (6.6)
Dispensing error	32 (2.9)

a Reasons for discontinuation are listed as reported by the investigator.

b Withdrawal of consent was due to treatment failure in 2 patients (1.8%).

c Includes discontinuations owing to protocol deviation (*n*=11), loss to follow-up (*n*=9), new cancer therapy (*n*=9; chronic myeloid leukemia (*n*=7) and endometrial cancer and non-Hodgkin lymphoma (*n*=1 each)), administrative problems (*n*=4) and death (*n*=4).

d Excluding periods of drug interruption.

e Defined as the sum of all doses administered divided by the time on treatment (including periods of drug interruption).

f Reasons for dosing error and scheduling conflict included commercial drug dispensed or delayed medication return by patient.

**Table 3:** Rates of MMR, MR^4^ and MR^4.5^ at 3, 12, 18 and 24 months (molecular analysis population; *n*=1052)

	*3 months*	*12 months*	*18 months*	*24 months*
MMR, *n* (%)	312 (29.7)	592 (56.3)	692 (65.8)	644 (61.2)
95% CI	26.9–32.4	53.3–59.3	62.9–68.6	58.3–64.2
MR^4^, *n* (%)	66 (6.3)	324 (30.8)	404 (38.4)	425 (40.4)
95% CI	4.8–7.7	28.0–33.6	35.5–41.3	37.4–43.4
MR^4.5^, *n* (%)	20 (1.9)	161 (15.3)	220 (20.9)	231 (22.0)
95% CI	1.1–2.7	13.1–17.5	18.5–23.4	19.5–24.5

Abbreviations: CI, confidence interval; MMR, major molecular response (*BCR-ABL1*^IS^⩽0.1%); MR^4^, molecular response 4 (*BCR-ABL1*^IS^⩽0.01%); MR^4.5^, molecular response 4.5 (*BCR-ABL1*^IS^⩽0.0032%).

**Table 4:** Nonhematological adverse events occurring in ⩾5.0% of patients at any grade or ⩾1% of patients at grade 3/4 (safety population; *N*=1089)

*Patients, n (%)*^a^	*Total (all grades)*	*Grade 2*	*Grade 3*	*Grade 4*
Rash	233 (21.4)	53 (4.9)	4 (0.4)	0
Pruritus	180 (16.5)	48 (4.4)	3 (0.3)	0
Headache	166 (15.2)	43 (3.9)	8 (0.7)	0
Abdominal pain	160 (14.7)	54 (5.0)	8 (0.7)	0
Fatigue	151 (13.9)	45 (4.1)	7 (0.6)	0
Nausea	123 (11.3)	37 (3.4)	5 (0.5)	0
Alopecia	115 (10.6)	15 (1.4)	1 (0.1)	0
Nasopharyngitis	113 (10.4)	28 (2.6)	0	0
Myalgia	99 (9.1)	20 (1.8)	3 (0.3)	0
Arthralgia	97 (8.9)	30 (2.8)	2 (0.2)	0
Asthenia	97 (8.9)	25 (2.3)	2 (0.2)	0
Diarrhea	94 (8.6)	22 (2.0)	2 (0.2)	0
Dry skin	93 (8.5)	19 (1.7)	0	0
Muscle spasms	93 (8.5)	13 (1.2)	0	0
Back pain	80 (7.3)	26 (2.4)	4 (0.4)	0
Constipation	67 (6.2)	21 (1.9)	1 (0.1)	0
Arterial hypertension	65 (6.0)	31 (2.8)	12 (1.1)	0
Vomiting	65 (6.0)	22 (2.0)	2 (0.2)	1 (0.1)
Cough	56 (5.1)	13 (1.2)	1 (0.1)	0
Insomnia	55 (5.1)	15 (1.4)	2 (0.2)	0
Pain in extremity	54 (5.0)	16 (1.5)	2 (0.2)	0

a Excluding events that started >28 days after last dose of study drug or month 24.

**Table 5:** Laboratory abnormalities (safety population; *N*=1089)^a^

	*Total (all grades)*	*Grade 2*	*Grade 3*	*Grade 4*
*Hematological laboratory abnormalities, n (%)*				
Anemia	774 (71.1)	116 (10.7)	15 (1.4)	0
Leukopenia	340 (31.2)	80 (7.3)	26 (2.4)	1 (0.1)
Lymphopenia	404 (37.1)	193 (17.7)	33 (3.0)	7 (0.6)
Neutropenia	208 (19.1)	71 (6.5)	32 (2.9)	20 (1.8)
Thrombocytopenia	438 (40.2)	51 (4.7)	41 (3.8)	24 (2.2)

*Biochemical laboratory abnormalities, n (%)*				
Alanine aminotransferase increase	659 (60.5)	41 (3.8)	24 (2.2)	3 (0.3)
Alkaline phosphatase increase	288 (26.4)	7 (0.6)	2 (0.2)	0
Amylase increase	177 (16.3)	24 (2.2)	15 (1.4)	0
Aspartate aminotransferase increase	356 (32.7)	15 (1.4)	10 (0.9)	1 (0.1)
Calcium decrease	326 (29.9)	37 (3.4)	1 (0.1)	12 (1.1)
Calcium increase	69 (6.3)	0	0	1 (0.1)
Creatinine increase	818 (75.1)^b^	44 (4.0)	1 (0.1)	0
Lipase increase	315 (28.9)	53 (4.9)	66 (6.1)	12 (1.1)
Magnesium decrease	124 (11.4)	2 (0.2)	2 (0.2)	2 (0.2)
Magnesium increase	97 (8.9)	0	16 (1.5)	1 (0.1)
Phosphate decrease	685 (62.9)	466 (42.8)	148 (13.6)	8 (0.7)
Potassium decrease	136 (12.5)	0	9 (0.8)	0
Potassium increase	147 (13.5)	31 (2.8)	7 (0.6)	6 (0.6)
Sodium decrease	137 (12.6)	0	3 (0.3)	0
Sodium increase	72 (6.6)	3 (0.3)	1 (0.1)	1 (0.1)
Total bilirubin increase	599 (55.0)	243 (22.3)	34 (3.1)	0
Uric acid increase	255 (23.4)	0	0	17 (1.6)

a Glucose, cholesterol and lipid monitoring was not mandated by the protocol, and therefore, the frequencies of these abnormalities was unknown.

b Creatinine elevations were grade 1 (defined as >1–1.5-fold baseline level or above the upper limit of normal to 1.5-fold the upper limit of normal^17^) in 71.0% of patients (*n*=773).
