# Supplementary material for: Frontline nilotinib in patients with chronic myeloid leukemia in chronic phase: results from the European ENEST1st study
Source: Leukemia. 2015 Nov 3;30(1):57–64. doi: 10.1038/leu.2015.270 (PMC4705425; doi:10.1038/leu.2015.270)
Supplement: Supplementary Information [file leu2015270x1.doc]

**Supplementary Appendix**

Supplement to: A Hochhaus, G Rosti, NCP Cross, et al. Frontline nilotinib in patients with chronic myeloid leukemia in chronic phase: results from the European ENEST1st study

Contents

[Supplemental Table 1. Study definitions of ischemic cardiovascular events 2](#__RefHeading___Toc422912908)

[Supplemental Table 2. Participating enrolling sites and principal investigators 3](#__RefHeading___Toc422912909)

[Supplemental Figure 1. Geographic distribution of treated patients 13](#__RefHeading___Toc422912910)

# Supplemental Table 1. Study definitions of ischemic cardiovascular events

|  | **Definition** |
| --- | --- |
| **PAD** | Any AE reported under the following PTs: aortic bypass, aortic embolus, aortic thrombosis, arterial occlusive disease, arterial stenosis, peripheral artery stenosis, peripheral artery thrombosis, peripheral artery bypass, peripheral artery stent insertion, peripheral artery restenosis, femoral artery occlusion, intermittent claudication, peripheral arterial occlusive disease, peripheral ischemia, peripheral vascular disorder, peripheral artery angioplasty, peripheral revascularization, ischemic limb pain, poor peripheral circulation, Raynaud phenomenon, celiac artery occlusion, splenic embolism, femoral artery embolism, hypothenar hammer syndrome, iliac artery embolism, iliac artery occlusion, peripheral arterial reocclusion, peripheral embolism, peripheral endarterectomy, popliteal artery entrapment syndrome, renal artery angioplasty, renal artery occlusion, renal artery thrombosis, renal embolism, subclavian artery embolism, subclavian artery occlusion, subclavian artery thrombosis, superior mesenteric artery syndrome, hepatic artery embolism, hepatic artery occlusion, hepatic artery thrombosis, mesenteric arteriosclerosis, mesenteric artery embolism, mesenteric artery stenosis, mesenteric artery stent insertion, mesenteric artery thrombosis, penile artery occlusion, and Leriche syndrome |
| **IHD** | Any AE reported under a PT in the narrow SMQ for ischemic heart disease |
| **ICVE** | Any AE reported under a PT in the narrow SMQ for ischemic cerebrovascular conditions or any of the following PTs: arteriogram carotid abnormal, intraoperative cerebral artery occlusion, retinal artery embolism, retinal artery thrombosis, and precerebral artery thrombosis |

Peripheral artery disease (PAD), ischemic heart disease (IHD), and ischemic cerebrovascular events (ICVEs) were defined as any adverse event (AE) reported under the Medical Dictionary for Regulatory Activities (MedDRA) preferred terms (PTs) or standardized MedDRA queries (SMQs) listed in the table. Ischemic cardiovascular events were defined as all AEs in the PAD, IHD, or ICVE groups.

# Supplemental Table 2. Participating enrolling sites and principal investigators

| **Country/site, city** | **Principal investigator(s)** |
| --- | --- |
| Austria |  |
| Universitätsklinik fur Innere Medizin, Innsbruck | Günther Gastl  Dominik Wolf |
| Hanuschkrankenhaus, Wien | Ernst Schloegl |
| LKH Graz Medizinische Universitätsklinik, Graz | Werner Linkesch  Albert Woelfler |
| Klinikum Wels - Grieskirchen GmbH, Wels | Josef Thaler |
| LKH Salzburg/Universität Klinikum der Paracelsus MPU, Salzburg | Richard Greil |
| LKH Rankweil, Rankweil | Alois Lang |
| Krankenhaus der Barmherzigen Schwestern Linz, Linz | Andreas Petzer |
| Krankenhaus der Stadt Wien Hietzing, Wien | Klaus Geissler |
| Belgium |  |
| Grand Hôpital de Charleroi (site Notre Dame), Charleroi | Delphine Pranger  Marc André |
| Centre Hospitalier du Luxembourg, Luxembourg | Sigrid Cherrier-De Wilde |
| Universitair Ziekenhuis Antwerpen, Edegem | Zwi Berneman |
| CHU Dinant Godinne UCL Namur – Dinant, Yvoir | André Bosly |
| Institut Jules Bordet, Brussels | Dominique Bron |
| AZ Delta, Roeselare | Dries Deeren |
| CHU Brugman, Laeken | André Efira |
| Hôpital Erasme, Brussels | Alain Kentos  Benjamin Bailly |
| UZ Gent, Gent | Lucien Noens |
| UZ Brussel, Brussels | Henri Schots |
| Jessa Ziekenhuis, Hasselt | Koen Theunissen |
| ASZ Aalst, Aalst | Joanna Van Erps |
| Centre Hospitalier Peltzer La Tourelle, Verviers | Gaetan Vanstraelen |
| UZ Leuven/Campus Gasthuisberg, Leuven | Gregor Verhoef |
| Hospital Universitaire St. Luc, UCL 5390, Brussels | Augustin Ferrant |
| AZ St.-Jan, Afdeling Oncologie, Brugge | Jan Van Droogenbroeck |
| Bulgaria |  |
| Multifunctional Hospital for Active Treatment “Sveta Marina,” Varna | KPI Dummy  Liana Gercheva-Kyuchukova |
| MHAT “Dr. George Stranski,” Pleven | Nikolay Tzvetkov |
| Multifunctional Hospital for Active Treatment “St. George,” Plovdiv | Emil Spasov |
| Alexandrovska University Hospital, Dermatology & Venerology, Sofia | Evgueniy Hadjiev |
| Specialized Hospital for Active Treatment of Haematological Diseases, Sofia | Georgi Mihaylov |

| **Country/site, city *(continued)*** | **Principal investigator(s) *(continued)*** |
| --- | --- |
| Croatia |  |
| University Hospital Centre Zagreb, Zagreb | Boris Labar |
| Clinical Hospital Center Rijeka, Rijeka | Antica Duletic-Nacinovic |
| Czech Republic |  |
| Fakultni nemocnice Hradec Králové, Hradec Králové | Jaroslava Voglova |
| Ustav hematologie a krevni transfuze, Praha | Hana Klamova |
| Faculty Hospital Olomouc, Olomouc | Edgar Faber |
| Fakultní nemocnice Brno – Bohunice, Brno | Jiri Mayer |
| Denmark |  |
| Aarhus University Hospital, Århus | Jesper Stentoft |
| Odense Universitets Hospital, Odense | Hanne Vestergaard |
| Rigshospitalet, Copenhagen | Ole Weis Bjerrum |
| Estonia |  |
| Oncology Clinic of Tartu University Hospital, Tartu | Ain Kaare |
| Finland |  |
| Hematologian tutkimusyksikkö, Helsinki | Kimmo Porkka |
| France |  |
| lnstitut Univ.du Cancer Toulouse-Oncopole, Toulouse | Francoise Rigal-Huguet |
| Hôpitaux de Chartres, Le Coudray | Lina Aljassem Abdelkader  Michel Maigre |
| Centre Hospitalier de la Côte Basque, Bayonne | Carla Araujo |
| Centre Hospitalier de Mâcon, Mâcon | Amine Belhabri |
| Centre Hospitalier de Limoges, Limoges | Dominique Bordessoule |
| Hopital Albert Michallon, Grenoble | Jean-Yves Cahn |
| CHU de Dijon, Dijon | Denis Caillot |
| Hopital Saint Vincent, Lille | Nathalie Cambier |
| GHIC Le Raincy-Montfermeil, Montfermeil | Yasmine Chait |
| Centre Hospitalier de la Région d’Annecy, Pringy | Pascale Cony-Makhoul |
| Centre Hospitalier de Chambéry, Chambéry | Maya Hacini  Selim Corm |
| Hôpital Nord Marseille, Marseille | Regis Costello |
| CHRU de Tours – Hôpital Bretonneau, Tours | Martine Delain |
| Hopital Robert Debre, Reims | Alain Delmer |
| Centre Hospitalier Sud Francilien, Corbeil-Essonnes | Bertrand Joly  Alain Devidas |
| Centre Hospitalier Général de Troyes, Troyes | Gérard Dine |
| Hopital Hotel Dieu, Nantes | Viviane Dubruille |
| Centre Hospitalier de Lens, Lens | Brigitte Dupriez |
| Centre Hospitalier de Saint-Quentin, Saint-Quentin | Reda Garidi |
| Hôpital Victor Dupouy, Argenteuil | Philippe Genet |
| Institut de Cancérologie de la Loire, Saint Priest en Jarez | Denis Guyotat |
| CHU de Clermont-Ferrand, Clemont-Ferrand | Marc Berger |
| **Country/site, city *(continued)*** | **Principal investigator(s) *(continued)*** |
| France *(continued)* |  |
| CHU de Brest – Hôpital Morvan, Brest | Jean-Christophe Ianotto |
| CHU de Nîmes, Nîmes | Eric Jourdan |
| CHU Jean Minjoz, Besancon | Fabrice Larosa |
| Centre Henri Becquerel- Centre de Lutte contre le Cancer de Haute-Normandie, Rouen | Pascal Lenain |
| Institut Bergonie, Bordeaux | Franҫois-Xavier Mahon |
| G H St Vincent-Clinique Ste Anne, Strasbourg | Fréderic Maloisel |
| HUS – Hôpital Civil, Strasbourg | Shanti Natarajan-Ame |
| Hôpital Victor Provo, Roubaix | Isabelle Plantier |
| CHRU de Montpellier – Hôpital Saint Eloi, Montpellier | Phillipe Quittet |
| Hopital Saint Louis, Paris | Delphine Rea |
| Centre Hospitalier de Blois, Blois | Philippe Rodon  Abderrazak El Yamani |
| Hopital A. Mignot, Le Chesnay | Philippe Rousselot |
| Centre Hospitalier William Morey, Chalon sur Saône | Bruno Salles |
| Centre Antoine Lacassagne, Nice | Antoine Thyss |
| CHU Henri Mondor, Creteil | Michel Tulliez |
| CHU d’Amiens – Hôpital Sud, Amiens | Jean-Pierre Marolleau  Ioana Vaida |
| Hopital Saint Antoine, Paris | Anne Vekhoff |
| Institut Paoli Calmettes, Marseille | Aude Charbonnier |
| CHD Les Oudairies, La Roche sur Yon | Bruno Villemagne |
| CHU Dunkerque, Dunkerque | Marc Wetterwald |
| Centre Hospitalier d’Avignon, Avignon | Hacene Zerazhi |
| Centre Hospitalier de Mulhouse – Hôpital Emile Muller, Mulhouse | Bernard Drenou |
| CHD Felix Guyon, Saint Denis | Philippe Agape |
| Germany |  |
| Charité Berlin Campus Virchow-Klinikum, Berlin | Philipp D. le Coutre |
| Ges. F. Medizinstatistik u. Projektentwicklung, Hannover | Michael Koenigsmann |
| Kliniken der Med. Hochschule Hannover, Hannover | Arnold Ganser |
| Universität Kiel – Medizinische Klinik, Kiel | Michael Kneba |
| Lübecker Onkologische Schwerpunktpraxis, Lübeck | Jens Kisro |
| Gemeinschaftspraxis Dr. Schick/Dr. Schmidt, München | Helmut Burkhar Schmidt |
| Universitätsklinikum Ulm, Ulm | Konstanze Döhner |
| Klinikum rechts der Isar der Technische Universität München, München | Justus Duyster  Philipp Jost |
| Universitätsklinikum Jena, Jena | Andreas Hochhaus |
| Gemeinschaftspraxis Drs Schlag & Schoettker, Würzburg | Rudolf Schlag |
| Klinikum der Universität Würzburg, Würzburg | Maria-Elisabeth Goebeler |

| **Country/site, city *(continued)*** | **Principal investigator(s) *(continued)*** |
| --- | --- |
| Germany *(continued)* |  |
| Universitätsklinikum Greifswald, Greifswald | Christian A. Schmidt  Gottfried Doelken |
| Praxis PD Dr. Jentsch-Ullrich, Magdeburg | Kathleen Jentsch-Ullrich |
| Gemeinschaftspraxis Dres. Illmer, Wolf, Jacobasch, Freiberg-Richter, Dresden | Thomas Illmer |
| Universitätsklinikum Frankfurt, Frankfurt | Oliver G. Ottmann |
| Universitätsmedizin Mannheim, Universität Heidelberg, Mannheim | Susanne Saussele |
| Johannes Gutenberg-Universität Mainz, Mainz | Thomas Kindler |
| Institut für Versorgungsforschung Onkologie, Koblenz | Rudolf Weide |
| Gemeinschaftspraxis Hämatologie/Onkologie Dres. Schliesser, Kaebisch, Giessen | Georg C. Schliesser |
| Onkologische Kooperation Harz, Goslar | Hans-Werner Tessen |
| Robert-Bosch-Krankenhaus, Stuttgart | Walter E. Aulitzky |
| Universitätsklinikum Essen gGmbH, Essen | Juergen Novotny |
| Evangelisches Krankenhaus, Hamm | Elisabeth Lange  Joerg Schubert |
| Universitätsklinikum Düsseldorf, Düsseldorf | Norbert Gattermann |
| Universitätsklinikum Köln, Köln | Christof Scheid |
| St. Antonius Hospital, Eschweiler | Frank Schlegel |
| Universitätsklinikum Aachen, Aachen | Tim H. Brümmendorf |
| HELIOS St. Johannes Klinik Duisburg, Duisburg | Aristoteles Giagounidis  Stephanie Groepper |
| Universitätsklinikum Bonn, Bonn | Viktor Janzen |
| Gemeinschaftspraxis Drs. Kalhori, Langer Nusch, Velbert | Arnd Nusch |
| Universitätsmedizin Rostock, Rostock | Christian Junghanss |
| Universitätsklinikum Carl Gustav Carus, Dresden | Rainer Ordemann |
| Universitätsklinikum Leipzig, Leipzig | Dietger Niederwieser |
| Universitätsklinikum Hamburg-Eppendorf, Hamburg | Philippe Schafhausen |
| Klinikum Lippe-Lemgo, Lemgo | Frank Hartmann |
| Onkologische Schwerpunktpraxis Bielefeld, Bielefeld | Marianne Just |
| Onkologische Schwerpunktpraxis Bottrop, Bottrop | Carla Hannig |
| Onkologische Gemeinschaftspraxis, München | Wolfgang Abenhardt |
| Kliniken Sindelfingen-Böblingen, Sindelfingen | Markus Ritter |
| Praxis Dr. Dietzfelbinger, Herrsching | Herrmann Dietzfelbinger |
| Onkologische Praxis Drs. Schwaner u. Tamm, Berlin | Ingo Tamm |
| Klinikum Bayreuth, Bayreuth | Alexander Kiani |
| Gemeinschaftspraxis Drs. Vehling-Kaiser und Greif, Landshut | Ursula Vehling-Kaiser |
| Praxis Drs. Blumenstengel, Eisenach | Klaus Blumenstengel |
| Klinikum Nürnberg Nord, Nürnberg | Christiane Falge |
| Internistische Praxisklinik Dr. Zimber, Nürnberg | Joachim Zimber |
| Universitätsklinikum Freiburg, Freiburg | Cornelius Waller |
| **Country/site, city *(continued)*** | **Principal investigator(s) *(continued)*** |
| Germany *(continued)* |  |
| Praxis Dr. Jakob, Offenburg | Andreas Jakob |
| Städtisches Klinikum München GmbH, München | Meinolf Karthaus |
| Gemeinschaftspraxis Drs. Eggert, Wiegand, Jehner, Moers | Joerg Wiegand |
| Praxis Dr. Kirsch und Dr. Kiewe, Berlin | Andreas Kirsch |
| Universitätsklinikum Giessen und Marburg GmbH, Giessen | Alexander Burchardt |
| Klinikum Kassel GmbH, Kassel | Sandra Tebbe |
| Bernward-Krankenhaus, Hildesheim | Ulrich Kaiser |
| Praxis Dr. Reich, Berlin | Gernot Reich |
| Gemeinschaftspraxis Drs. Sandherr, Perker, Weilheim | Michael Sandherr |
| Universitätsklinikum Münster, Münster | Steffen Koschmieder |
| Onkologische Gemeinschaftspraxis Dr. Cordes, Frankfurt | Hans-Jörg Cordes |
| Gemeinschaftspraxis Dr. Soeling, Dr. Siehl, Dr. Hirschmann, Kassel | Ulrike Soeling |
| Praxis Dr. Walter, Paderborn | Michael Walter |
| Gemeinschaftspraxis Drs.Rudolph, Sengpiel, von Verschuer, Essen | Ulla von Verschuer |
| Katholisches Krankenhaus Hagen GmbH, Hagen | Hans-Walter Lindemann |
| Onkologische Gemeinschaftspraxis Rems-Murr, Schorndorf | Dieter Buerkle |
| Praxis Dr. Reiter, Viersen | Wilhelm W. Reiter |
| Diakonie Krankenhaus Schwäbisch-Hall gGmbH, Schwäbisch-Hall | Thomas Geer |
| Gemeinschaftspraxis Kamp, Wendlingen | Torsten Kamp |
| Onkologische Gemeinschaftspraxis, Dr. Decker, Ravensburg | Thomas Decker |
| St Vincentius-Krankenhaus, Karlsruhe | Joerg Mezger |
| Franziskus-Hospital Mönchengladbach Maria Hilf GmbH, Mönchengladbach | Ullrich Graeven |
| Gemeinschaftspraxis Drs Sieg, Schröder, Brouwers, Mülheim | Katharina Sieg |
| Klinikum Traunstein, Traunstein | Thomas Kubin |
| Zentralklinikum Augsburg, Augsburg | Christoph Schmid |
| Klinikum Fulda, Fulda | Andrea Distelrath |
| Asklepios Klinik Triberg, Triberg | Gerhard Adam |
| Klinikum Frankfurt/Oder, Frankfurt (Oder) | Wolfgang Stein |
| Hämatologisch-Onkologische Praxis, Neunkirchen | Peter Schmidt |
| Klinikum Bamberg, Bamberg | Roland Repp |
| Klinikum Bremen Mitte, Bremen | Bernd Hertenstein |
| Greece |  |
| General Hospital of Athens “Laiko,” Athens | Panayiotis Panayiotidis |
| General Hospital of Thessaloniki “Hippokrateio,” Thessaloniki | Ioannis Klonizakis |
| General Hospital of Athens G. Gennimatas, Athens | Nikolaos Anagnostopoulos |
| University General Hospital of Heraklion, Heraklion | Helen Papadaki |
| University General Hospital of Larissa, Larissa | Panagiota Matsouka |
| **Country/site, city *(continued)*** | **Principal investigator(s) *(continued)*** |
| Greece *(continued)* |  |
| University General Hospital of Patras, Patras | Alexandros Spyridonidis  Argiris Symeonidis |
| Hungary |  |
| Egyesitett Szent Istvan-Szent Laszlo Korhaz-Rendelointezet, Budapest | Tamas Masszi |
| Szent-Györgyi Albert Klinikai Központ, Szeged | Zita Borbenyi |
| Debreceni Egyetem Klinikai Centrum, Debrecen | Miklos Udvardy |
| University of Pécs, Pécs | Marianna David  Agnes Nagy |
| Kaposi Mór Oktató Kórház, Kaposavár | Miklos Egyed |
| Italy |  |
| A.O. Universitaria Pisana – Pres. Osp. Santa Chiara – Università degli Studi, Pisa | Mario Petrini |
| Presidio Osp. A. Perrino – ASL BR, Brindisi | Angela Melpignano  Giovanni Quarta |
| A.O. di Rilievo Nazionale A. Cardarelli, Napoli | Felicetto Ferrara |
| A.O. Niguarda Ca’ Granda, Milano | Ester Pungolino |
| A.O. Spedali Civili di Brescia Università degli Studi, Brescia | Giuseppe Rossi |
| Ospedale Oncologico A. Businco, Cagliari | Emanuele Angelucci |
| Fondazione IRCCS Ca’ Granda Ospedale Maggiore Policlinico Università Studi, Milano | Giorgio Lambertenghi Deliliers  Alessandra Iurlo |
| A.O. di Reggio Emilia – Arcispedale S. Maria Nuova, Reggio Emilia | Paolo Avanzini |
| A.O. Universitaria Consorziale Policlinico di Bari Università degli Studi, Bari | Giorgina Specchia |
| IRCCS A.O. Universitaria San Martino-IST Istituto Nazionale per la Ricerca sul Cancro, Genova | Marco Gobbi |
| A.O. Univ. Ferrara – Arcispedale S. Anna Università degli Studi, Cona | Antonio Cuneo |
| A.O. Santa Maria di Terni – Università Studi Perugia, Temi | Anna Marina Liberati |
| A.O. Universitaria Senese Ospedale Santa Maria alle Scotte, Siena | Francesco Lauria  Monica Bocchia |
| IRCCS Casa Sollievo della Sofferenza, San Giovanni Rotondo | Nicola Cascavilla |
| Presidio Ospedaliero Nicola Giannattasio, Rossano | Francesco Iuliano |
| Stab. Ospedaliero Umberto I – PO Nocera-Pagani – ASL Salerno, Nocera Inferiore | Alfonso Maria D’Arco  Paolo Danise |
| Presidio Ospedaliero Roberto Binaghi – ASL Cagliari, Cagliari | Giovanni Caocci |
| Presidio Ospedaliero Vito Fazzi ASL Lecce, Lecce | Nicola Di Renzo |
| IRCCS A.O. Universitaria San Martino-IST Istituto Nazionale per la Ricerca Cancro, Genova | Angelo Michele Carella |
| Casa di Cura di Alta Specialità La Maddalena – Dipartimento Oncologico di III Livello, Palermo | Maurizio Musso |
| Policlinico Universitario A. Gemelli-Università Cattolica del Sacro Cuore, Roma | Simona Sica  Giuseppe Leone |
| **Country/site, city *(continued)*** | **Principal investigator(s) *(continued)*** |
| Italy *(continued)* |  |
| Presidio Ospedaliero Livorno – ASL Livorno 6, Livorno | Enrico Capochiani |
| Ospedale S. Eugenio ASL Roma C, Roma | Elisabetta Abruzzese |
| A.O. Ospedale San Salvatore, Pesaro | Giuseppe Visani |
| Presidio Ospedaliero Spirito Santo-AUSL Pescara Università Studi, Pescara | Giuseppe Fioritoni  Paolo Di Bartolomeo |
| A.O. di Padova-Universtà degli Studi, Padova | Gianpietro Semenzato |
| Osp S. Maria della Misericordia- A.O. Perugia – Università Studi, Perugia | Franca Falzetti |
| A.O. Universitaria di Sassari, Sassari | Simonetta Maria Pardini  Maurizio Longinotti |
| A.O. S.G. Moscati, Avellino | Fausto Palmieri  Nicola Cantore |
| A.O. di Bologna Policl. S. Orsola-Malpighi Università degli Studi, Bologna | Michele Baccarani  Michele Cavo |
| A.O. Universitaria S. Giovanni Battista di Torino, Torino | Patrizia Pregno |
| Presidio Ospedaliero S. Maria delle Croci AUSL Ravenna, Ravenna | Alfonso Zaccaria  Roberto Zanchini |
| A.O. Universitaria Maggiore della Carità, Novara | Gianluca Gaidano |
| A.O. Bianchi Melacrino Morelli – Presidio Ospedali Riuniti, Reggio Calabria | Francesco Nobile  Bruno Martino |
| Stab. Ospedale S. Giuseppe Moscati- PO Centrale SS Annunziata, Taranto | Patrizio Mazza  Alessandro Maggi |
| A.O. Pugliese-Ciaccio, Catanzaro | Luciano Levato  Stefano Molica |
| IRCCS A.O. Universitaria San Martino-IST Istituto Nazionale per la Ricerca sul Cancro, Genova | Francesco Frassoni  Andrea Bacigalupo  Maria Teresa Van Lint |
| A.O. S Croce e Carle, Cuneo | Davide Rapezzi  Andrea Gallamini |
| A.O. Policlinico Umberto I – Università La Sapienza, Roma | Giuliana Alimena |
| Azienda Sanitaria Ospedaliera Universitaria San Luigi Gonzaga Orbassano Università Studi Torino, Orbassano | Giuseppe Saglio |
| A.O. Universitaria Policlinico Federico II Università degli Studi, Napoli | Fabrizio Pane |
| A.O. Universitaria Policlinico di Modena Università Studi Modena e R. Emilia, Modena | Roberto Marasca |
| Fondazione IRCCS Policlinico San Matteo Università degli Studi Pavia, Pavia | Ester Maria Orlandi |
| A.O. Universitaria Careggi-Università Studi di Firenze, Firenze | Alberto Bosi |
| Complesso Ospedaliero di Belcolle, Ronciglione | Marco Montanaro |
| Pres. Ospedaliero Santa Maria di Ca’ Foncello, Treviso | Filippo Gherlinzoni |
| A.O. San Carlo, Potenza | Attilio Olivieri  Michele Pizzuti |
| A.O. Universitaria Ospedali Riuniti Umberto I-GM Lancisi-G Salesi-Università Studi, Ancona | Pietro Leoni |

| **Country/site, city *(continued)*** | **Principal investigator(s) *(continued)*** |
| --- | --- |
| Italy *(continued)* |  |
| Presidio Ospedaliero Cesare Zonchello ASL 3 Nuoro, Nuoro | Anna Lisa D. Noli  Attilio Gabbas |
| Latvia |  |
| National Centre of Haematology, Riga | Sandra Lejniece |
| Lithuania |  |
| Vilnius University Hospital, Santariskiu Klinikos, Vilnius | Laimonas Griskevicius |
| Klaipeda Republican Hospital, Klaipeda | Mindaugas Jurgutis |
| The Netherlands |  |
| Vrije Universiteit Medical Centre, Amsterdam | G.J. Ossenkoppele |
| Erasmus Medisch Centrum (Daniel Den Hoed Kliniek), Rotterdam | J.J. Cornelissen |
| University Medical Center St. Radboud, Nijmegen | A. Schattenberg |
| Reinier de Graaf Gasthuis, Delft | Ward Posthuma |
| Medische Spectrum Twente, Enschede | W.M. Smit |
| Isala Klinieken, locatie Sophia, Zwolle | M. Van Marwijk Kooy |
| Universitair Medisch Centrum Groningen, Groningen | E. Vellenga |
| Norway |  |
| Rikshospitalet, Oslo | Tobias Gedde-Dahl |
| Haukeland University Hospital, Bergen | Bjoern Tore Gjertsen |
| St. Olafs Hospital HF Trondheim, Trondheim | Henrik Hjorth-Hansen |
| Helse Stavanger HF, Stavanger universitetssykehus, Stavanger | Mohammed Waleed Majeed |
| Universitetssykehuset i Nord-Norge, Tromsø | Anders Vik |
| Poland |  |
| Klinika Hematologii AM, Gdansk | Andrzej Hellmann |
| Instytut Hematologii i Transfuzjologi, Warszawa | Krzysztof Warzocha |
| Klinika Hematologii, Nowotworow Krwi i Transplantacji Szpiku, Wroclaw | Kazimierz Kuliczkowski |
| Akademia Medyczna w Lublinie, Lublin | Anna Dmoszynska  Malgorzata Kowal |
| Malopolskie Centrum Medyczne, Krakow | Aleksander Skotnicki |
| MTZ Clinical, Warsaw | Wieslaw Wiktor-Jedrzejczak |
| Portugal |  |
| Centro Hospitalar de Lisboa Central EPE Hospital Santo Antonio dos Capuchos, Lisboa | Aida Botelho de Sousa |
| Instituto Portugues de Oncologia do Porto, Porto | Ana Espirito Santo |
| Romania |  |
| Institutul Clinic Fundeni, Bucharest | Daniel Coriu |
| Spitalul Clinic Coltea, Bucharest | Anca Roxana Lupu |
| Oncology Institute I. Chiricuta, Cluj-Napoca | Ljubomir Petrov |
| Emergency Municipal Clinical Hospital, Timisoara | Hortensia M. Ionita |
| Spitalul Clinic Judetean de Urgente Sfantul Spiridon, Iasi | Cristina Burcoveanu |
| Spitalul Universitar de Urgenta Bucuresti, Bucharest | Horia Bumbea |
| **Country/site, city *(continued)*** | **Principal investigator(s) *(continued)*** |
| Slovakia |  |
| Fakultna nemocnica L. Pasteura, Kosice | Natalia Stecova  Elena Tothova |
| Martinsk. Fakultna Nemocnica, Martin | Emilia Flochova |
| FNsP Bratislava, Nemocnica sv. Cyrila a Metoda, klin. Hema, Bratislava | Martin Mistrik |
| Slovenia |  |
| University Medical Centre Ljubljana, Ljubljana | Irena Preloznik Zupan |
| General Hospital Celje, Celje | Mateja Grat |
| Spain |  |
| Hospital Universitari Germans Trias I Pujol, Badalona | Blanca Xicoy Cirici |
| Hospital de la Santa Creu i Sant Pau, Barcelona | Anna Sureda Balari  Jorge Sierra Gil |
| Hospital del Mar, Barcelona | Alberto Alvarez Larran |
| Hospital Vall D’Hebron, Barcelona | Isable Massague Ferrer  Pere Barba Sunol |
| Hospital Universitario Doctor Negrin, La Palmas de Gran Canaria | Maite Gomez Casares |
| Hospital Clinico Universitario de Valencia, Valencia | Juan Carlos Hernandez Boluda |
| Hospital Universitario Basurto, Bilbao | Jose Maria Beltran de Heredia Oya  Fernando Marco de Lucas |
| Hospital Universitario Miguel Servet, Zaragoza | Pilar Giraldo Castellano |
| Hospital de Cruces, Baracaldo | Juan Carlos Garcia Ruiz |
| Hospital Marques de Valdecilla, Santander | Eulogio Conde Garcia |
| Hospital Clinic l Provincial De Barcelona, Barcelona | Francisco Cervantes Requena |
| Complejo Hospitalario de Navarra, Pamplona | Eduardo Olavarria Lopez |
| Hospital Universitario Joan XXIII, Tarragona | Rolando Omar Vallansot |
| Hospital Morales Meseguer, Murcia | Francisca Ferrer Marin  Virginia Perez Andreu |
| Hospital General Universitario de Elche, Elche | Venancio Conesa Garcia |
| Hospital de Donostia, San Sebastian | Maria Asuncion Echeveste Gutierrez |
| Hospital Universitario de Canarias, Santa Cruz de Tenerif | Sunil Lakhwani Lakhwani |
| Complejo Hospitalario de Orense, Orense | Jose Luis Sastre Moral |
| Complejo Uni. Hosp. A Coruna (antes Hospital Juan Canalejo), La Coruna | Javier Batlle Fondorona |
| Hospital Universitario de Salamanca, Salamanca | Consuelo Del Canizo Fernandez Roldán |
| Hospital Universitario de la Princesa, Madrid | Juan Luis Steegmann |
| Hospital Virgen de la Salud, Toledo | Felipe Casado Montero |
| Hospital Ramon Y Cajal, Madrid | Jose Valentin Garcia Gutierrez |
| Fundacion Jimenez Diaz, Madrid | Jose Luis Lopez Lorenzo |
| Hospital Clinico San Carlos, Madrid | Ascension Pena Cortijo |
| Hospital 12 de Octubre, Madrid | Maria Liz Paciello Coronel |
| Hospital La Paz, Madrid | Raquel De Paz Arias |
| **Country/site, city *(continued)*** | **Principal investigator(s) *(continued)*** |
| Spain *(continued)* |  |
| Hospital General de Asturias, Oviedo | Angel Ramirez Payer |
| Hospital Puerta de Hierro, Majadanonda | Guiomar Bautista Carrascosa |
| Hospital Mutua de Terrassa, Terrassa | Ferran Vall Llovera Calmet |
| Hospital Clinico Universitario Lozano Blesa, Zaragoza | Luis Palomera Bernal |
| Sweden |  |
| Karolinska Universitetssjukhuset Solna, Stockholm | Leif Stenke |
| Sahlgrenska Universitetssjukhuset, Göteborg | Hans Wadenvik |
| Akademiska sjukhuset, Uppsala | Bengt Simonsson  Ulla Olsson Stroemberg |
| Karolinska Universitetssjukhuset, Huddinge | Soren Lehmann |
| Norrlands Universitetssjukhus, Umeå | Berith Markevaern |
| Skånes Universitetssjukhus, Lund | Johan Richter |
| Sunderby sjukhus, Luleå | Kristina Myhr Eriksson |
| Universitetssjukhuset, Linköping | Kourosh Lotfi |
| Switzerland |  |
| Hôpitaux universitaires de Genève, Genève | Yves Chalandon |
| Inselspital Bern, Bern | Gabriela Baerlocher |
| Raetisches Kantons- und Regionalspital Chur, Chur | Dirk L. Kienle |
| Universitatsspital Zürich, Zürich | Gayathri Nair |
| United Kingdom |  |
| Hammersmith Hospital, London | Dragana Milojkovic  David Marin |
| Kings College Hospital, London | Aloysius Ho  Ghulam Mufti  Hugues De Lavallade |
| University Hospital of Wales, Cardiff | Jonathan Kell |
| Nottingham City Hospital, Nottingham | Jenny Byrne |
| The Dudley Group Of Hospitals NHS Trust, Dudley | David Bareford  Savio Fernandes |
| Churchill Hospital Department Oncology/Haematology, Oxford | Adam Mead  Paresh Vyas |
| Hillingdon Hospitals NHS Trust, Uxbridge | Richard Kaczmarski |

A.O., azienda ospedaliera; ASL, azienda sanitaria locale; AZ, Algemeen Ziekenhuis; CHD, centre hospitalier départemental; CHR, centre hospitalier régional; CHRU, centre hospitalier regional universitaire; CHU, centre hospitalier universitaire; EPE, Entidades Públicas Empresariais; gGmbH, gemeinnützige Gesellschaft mit beschränkter Haftung; GHIC, Groupe Hospitalier Intercommunal; HF, helseforetak; HUS, University Hospitals of Strasbourg; IRCCS, istituto di ricovero e cura a carattere scientifico; LKH, Landeskrankenhaus; MPU, paracelsus medizinische privatuniversität; NHS, National Health Service; UCL, Université catholique de Louvain; UZ, Universitair Ziekenhuis.

# Supplemental Figure 1. Geographic distribution of treated patients (intent-to-treat population; N = 1089).


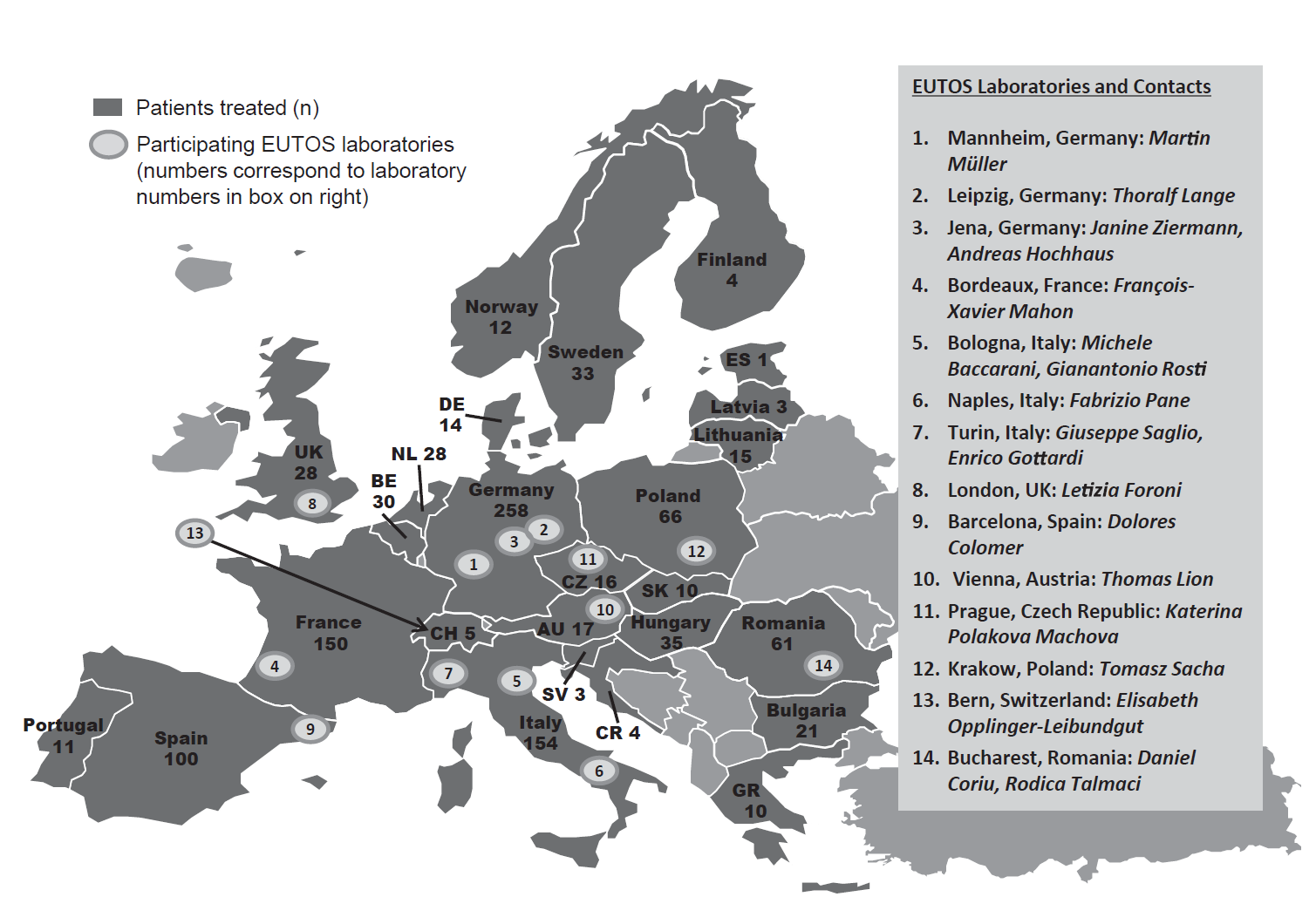


AU, Austria; BE, Belgium; CH, Switzerland; CR, Croatia; CZ, Czech Republic; DE, Denmark; ES, Estonia; EUTOS, European Treatment and Outcome Study; GR, Greece; NL, the Netherlands; SK, Slovakia; SV, Slovenia.
